# The role of socio-demographic variables and buying habits in determining milk purchasers’ preferences and choices

**DOI:** 10.3389/fnut.2023.1072208

**Published:** 2023-02-08

**Authors:** Valentina Maria Merlino, Oriana Mosca, Simone Blanc, Antonina Sparacino, Stefano Massaglia, Danielle Borra, Giulia Mastromonaco, Ferdinando Fornara

**Affiliations:** ^1^Department of Agricultural, Forest and Food Sciences, University of Turin, Turin, Italy; ^2^Department of Education, Psychology, Philosophy, University of Cagliari, Cagliari, Italy

**Keywords:** socio-demographic variables, purchasing habits, cow milk, stated preferences, best-worst scaling

## Abstract

Emerging new purchasing behaviors have been reflected in the sales trends of dairy products, mainly in cow milk consumption. This study aimed to investigate the preferences of milk purchasers toward different product attributes, by considering both individuals’ socio-demographic characteristics (SD) and milk purchasing habits (PH) as independent variables in the milk consumption model definition. To achieve this objective, a questionnaire was administered to a sample of 1,216 residents in Northwest Italy. The application of the Best-Worst scaling (BWS) methodology to define the purchasers’ declared preferences toward a set of 12 milk attributes, showed that milk origin and expiry date are the most important attributes for milk choice in the decision-making process. The correlation analysis showed that the SD and milk purchasing habits variables affect the definition of stated preferences heterogeneously between the intrinsic, extrinsic, and credence attributes.

## 1. Introduction

The orientation of choice and purchase of cow’s milk, both in terms of quantity and quality, has undergone considerable changes in recent years. Consumer choice has varied in the types of milk chosen, as well as modeled the weight and importance given to the individual attributes that define the product. These changes have been influenced, first and foremost, by the evolving needs of the modern consumer, who is increasingly oriented toward the inclusion of healthy and sustainable (environmentally, socially, and economically friendly) food styles (1–3). The re-orientation of purchase behavior focused on ecocentrism is visible for many food products that are believed to harm the environment or human health, such as cow’s milk. In the collective imagination, on the other hand, consumers have developed a negative conception of cow’s milk, considering it to be an unhealthy product (4), unsuitable for increasingly intolerant consumers, and deriving from production systems that are unsustainable for the environment and society (5–8). In the Italian context, in the last decade, cow milk consumption has suffered from negative trends (9, 10). Specifically, the COVID-19 pandemic event led the Italian population to change consumption habits, e.g., preferring products of Italian tradition such as milk, butter, and cheese (11). With the partial return to normality and the reopening of the so-called “HoReCa” (Hotel, Restaurant, Café) channel, however, the dairy sector saw a decrease in consumption shares compared to 2019, i.e., the year of the positive downturn dictated by the lockdown (12).

During the complex decision-making process, individuals simultaneously assess the intrinsic and qualitative attributes of the product, together with the values conveyed by the product itself, such as environmental protection, social value, safety, and superior quality defined by the link with the land or the short supply chain (13, 14). These latter aspects, on the other hand, have led to a reversal in the preferences of milk purchasers and consumers who seem to be choosing “conventional” cow’s milk again, provided that it is a more socially, economically, and environmentally sustainable product in terms of being local, qualitatively certified and organic milk (15). In fact, the aspects that still bind a large proportion of consumers to cow’s milk consumption are those related to the milk origin and the support for small-scale dairy production by farmers, which is supposed to enhance the local territory (8, 16).

On the other hand, the Italian dairy sector is characterized by the presence of many small, family run farms, distributed mainly in marginal areas and based on traditional and environmentally sustainable production systems (3, 8). Therefore, given the needs of modern consumers, together with their willingness to pay a premium price for more sustainable and local milk (11), the composition of the national milk supply seems to present several elements of enhancement and competitiveness akin to recovery and growth of the whole sector at the national level.

Product attributes evaluation during the choice-making process is influenced, in addition, by the individuals’ socio-demographic characteristics (SD) and milk purchasing habits (PH) (16). Hence, there is a need to examine their specific weight more in-depth. Regarding SD characteristics, there is evidence of their impact on food choices, in particular for products linked to sustainability and health concerns (12, 14, 17–19). In Akpinar et al. (12), for example, it seems that there were gender-determined differences in the perception of the organoleptic and aesthetic characteristics of fruit and vegetables, while the individuals’ level of education and income affected the evaluation of price, seasonality, and organic certification of the product. In particular, the higher education level of individuals was found to be the characteristic most likely to be associated with a pattern of choosing fresher, organic fruits and vegetable. In a more recent study (14), in which the effect of the variables gender, age, level of education, personal financial situation and number of children in the family on the ecological purchasing behavior of consumers was investigated, it was found that women had a more positive attitude toward the purchase of green products (i.e., organic food) than men, while young subjects were the most disbelieving toward ecological products.

In the case of milk consumption, (16) showed that SD characteristics affect the choice of fresh and Ultra High Temperature (UHT) milk: in particular, it was found that consumers of UHT milk were younger, less well-off economically, but better educated than those of fresh pasteurized milk. At the same time, women in modern society are often responsible for food purchasing for the family, making choices oriented to guarantee the wellness and the security of children and considering, together, the product convenience (20). In these terms, women/mothers often make choices based on the evaluation of product brand and product nutritional quality and origin (21–24).

Consumer profiles most likely to buy organic milk and branded conventional milk are characterized by individuals having high incomes, belonging to large households, and old age (25).

In addition, the preferences and attitudes toward a product are defined by individuals’ purchasing habits and the place of food purchase (26–28). For example, in the case of milk, Tabacco et al. (15) found that the different purchasing profiles defined in the research (based on stated preferences) distinguished themselves by their milk purchasing habits (in terms of the type of milk usually chosen: UHT, fresh pasteurized or both). For example, in the research conducted by (29) it seems that consumers of organic foods, selected especially for health reasons, prefer to buy these products directly from the producers, followed by supermarkets, specialized shops, and pharmacies. In another study, it seems that purchasers reoriented their food choices toward local products instead of buying directly from the producers (30). In addition, (31, 32) found that product quality and sustainability were the core element for consumers to buy directly from producers. In (33) the milk purchasing at milk vending machines was majorly linked to the consumer with a higher sensitivity toward environmental concerns, compared to the individuals buying milk at the supermarket. However, the Italian consumer buys cow’s milk especially in the large retail distribution (9, 15), basing their choices specifically on the product brand and convenience (34).

To our knowledge, no research has explored the specific impact of each single SD variable and of the place of milk purchase on the preference level toward a set of quality attributes that describe cow’s milk. To fill this gap, the aim of this research was to verify the weight and the role of individuals’ SD characteristics and PH (in terms of the place of milk purchase) on the preference index calculated for each selected descriptor of cow’s milk, i.e., credence, intrinsic, and extrinsic attributes of individuals’ choice (15), to predict different milk purchasing patterns in a specific geographic area of Italy. This selection was made by considering the literature regarding the attributes of milk, and food products in general, that drive consumer preferences when making purchasing choices (30, 34–37). Specifically, credence attributes are those whose veracity cannot be tested during either the purchasing or the consumption process. The purchaser must therefore rely on information conveyed by the media, the label, advertising, and other sources (38). The latter is, for instance, product certifications, e.g., related to product sustainability and ethics, and local origin (39). Regarding intrinsic attributes, they are objectively measurable product characteristics that relate to the physical appearance of the product. Intrinsic attributes of cow milk that influence purchasing decisions are for instance those related to nutritional value, product taste, and fat content. On the other hand, extrinsic attributes are an integral part of the product, related to the product’s appearance such as brand name or price but are not physical characteristics.

Accordingly, we formulated the following hypotheses:

H1: SD characteristics have an impact on the individuals’ stated preferences toward cow’s milk attributes; in particular, we expect that gender, age, level of education, and income have an impact on the evaluation of belief attributes, while gender and presence of children on the evaluation of intrinsic and extrinsic quality attributes of cow’s milk.

H2: PH affects the decision-making process of cow’s milk purchase.

## 2. Materials and methods

### 2.1. Participants, procedure, and tools

The survey was carried out randomly by selecting volunteer participants from outside the points of sale of large-scale retail in Northwest Italy and respondents who had answered the online version of the questionnaire. The considered area, selected both for the face-to-face interviews and the online survey, plays an important role in the national production and purchasing phases in the cow’s milk sector. In fact, in 2021, 54.2% of the milk produced in Italy came from this area, while it was households in Northwest Italy who provided 28% of the contribution to milk and dairy product costs (10). The eligibility criteria of the interviewees were as follows, in accordance with those already used in Tabacco et al. (15): a minimum age of 18 years and being responsible for purchasing milk for personal consumption or other household members. These criteria were verified before the questionnaire administration through preliminary questions and after informed consent. Data were collected from October to December 2020. A choice experiment was conducted through a structured questionnaire submitted to respondents either face-to-face or online.

The questionnaire, which follows the ethical standards defined by the Declaration of Helsinki, was developed in Italian and approved by the University Bioethics Committee of the University of Turin.^1^

The questionnaire included three main sections, addressing respectively: SD characteristics, i.e., age, gender, family size, occupation, educational level, and average annual income of the family; PH, i.e., the place of milk purchase (supermarket, convenience stores, discounts, and open market/producer); and finally, the Best-Worst scaling (BWS) questions scheme (Supplementary material).

The Best-Worst scaling approach belongs to the discrete choice methodologies and allows for a trade-off of preferences between elements. This method was introduced (by Finn and Louviere) and formalized (by Marley and Louviere) between 1992 and 2005 (40, 41). The economic analysis of discrete choices, as well as the BWS, is based on the use of the random utility maximization (RUM) model, which allows one to directly estimate the declared preference degree of a subject or a population toward a set of attributes that describe a product. During the choice experiment, by repetitively asking the respondents to choose the best and the worst alternatives for each BWS question (choice set) (Table 1), it is possible to calculate a mean preference index for each attribute obtained considering the sample size, using a probabilistic approach. This preference index is a quantitative score (average raw score) that could be used to create a preference ranking obtained from the individual levels of preference declared by the respondents, which were then defined and assigned to each considered qualitative attribute (42). Our BWS experimental design, developed using the Sawtooth MaxDiff Designer software (SSI-version 8.4.6, Orem, UT, the USA)^2^ was created following the commonly used Balanced Incomplete Block scheme: given a set of n attributes, r choice sets are provided, each containing t attributes (constant condition *n* > t), according to a balanced incomplete block scheme (43). Therefore, each attribute appears s times in the experimental design and each couple of items appears α times. The α and s numbers are integers, and α can be calculated with the α = s × (*t*–1)/(*n*–1) equation (44, 45). In particular, our framework was structured as follows: starting from a selection of 12 attributes, the questionnaire contained nine choice sets (Best-Worst scaling questions), each comprising four attributes, presented in four different versions of the questionnaire (to increase the combination of attributes in the sets). The selected attributes included credence attributes (organic certification, local origin certification, and high-quality certification), intrinsic attributes (nutritional value, taste, and fat content–skimmed, semi-skimmed or whole), and extrinsic attributes (brand, label information, indication of origin–national or international–expiration date, price, and packaging material–plastic jug, cardboard carton, glass) (see Table 2).

**TABLE 1:** Example of a best-worst scaling question (choice set).

Least important (only one choice)	Milk attributes	Best important (only one choice)
○	Expiration date	○
○	Price	○
○	Fat content	○
○	Packaging material	○

Indicate the most important (BEST) and the least important (WORST) attributes during the milk choice.

**TABLE 2:** Attributes analyzed with the Best-Worst scaling methodology.

Attributes category	The selected milk attributes
Credence attributes	Organic certification
	Locally farmed
	High-quality certification
Intrinsic attributes	Fat content (skim, partially skimmed, and whole)
	Taste
	Nutritional value
Extrinsic attributes	Brand
	Label claims (visual and verbal)
	Origin indication (national/abroad)
	Expiry date
	Price
	Package type (plastic jug, cardboard carton, glass)

### 2.2. Data analysis

For each item of the questionnaire, respondents were asked to choose the most important (BEST) and the least important (WORST) element underlying their choice, i.e., the maximum difference pair. Starting from the feedback collected from the data collection they were analyzed using the Sawtooth software (SSI version 8.4.6, Orem, UT, USA; see text footnote 2). The count ratio and the Bayes hierarchical estimation (HB) were obtained for the evaluation of the stated preferences. The Hierarchical Bayes model was employed to calculate in a probabilistic way the average preference score for each attribute that was selected for the choice experiment. HB analysis is considered by some to be the gold standard for estimating individual-level utility values in best-worst scaling experiments as it can account for population-level utilities when estimating individual-level utilities, which may yield more precise estimates. In particular, starting from the count ratio (the number of times the single attribute was selected as best: COUNTbest) and the number of times it was selected as worst: COUNTworst), the software provides an aggregate value of preference per single attribute obtained based on the sample size (average raw score or A-RS, or B-W scores). Specifically, the A-RS is defined by the difference between COUNTbest and COUNTworst, related to the sample size multiplied by the r (i.e., the number of times the single attribute appears in the questionnaire) that in our case was equal to 3 (15, 16). The responses to all the attribute subsets collected with the BWS approach are often analyzed using a Hierarchical Bayes framework, a random utility theory approach that is based on the method of paired comparisons (46). The underlying assumption is that the utility or value given to alternative A over alternative B is indicated by the frequency with which A is selected over B. The greater the number of times A is selected as BEST, compared to B (as WORST), the greater the preference for A over B (47). In addition to obtaining a ranking, the result is a scale of importance or maximum distance between two alternatives. Applying this principle to a set of items, it is assumed that each individual has a personal rank that determines his or her choices and that the utility assigned to each alternative represents the individual item’s position within its scale (48).

We chose this approach following the suggestion of Jaeger et al. (49) that highlighted that preference data elicited using best–worst scaling may better enable the discovery of differences in sample preferences, in comparison to other statistical approaches such as logistic regression. In fact, HB models have recently been shown to outperform aggregate methods (MNL) and latent class methods in estimations of B-W choice data related to food quality attributes (50, 51). Lagerkvist et al. (51), specifying, in addition: “HB models can handle the presence of within- and between-respondent choice heterogeneity and offer the advantage of investigating the probability distribution of the parameters given the data, instead of the opposite as in random parameters logit models (RPL), which means that data quality is not lost in estimating a HB model. A further advantage of HB is the ability to generate individual-specific data from sparse data sets.”

In addition, both (51, 52) showed that MNL parameter estimates are proportional to B-W scores. This is in agreement with Marley and Louviere who presented mathematical evidence showing that the scale values of a set of items, derived through MNL modeling of best-worst choices, can be fully approximated through the simplest difference score analysis (i.e., Best-minus-Worst scores and the A-RS) (41). After data analysis, the software provides the values of A-RSs, obtained for each attribute, and a new matrix of data composed of a number of rows equal to the sample size and 13 columns (12 for the attributes and 1 for the fit statistic value). For each row (subject), the individual value of preferences (preferences B-W index) for each attribute is indicated in the cell. The A-RSs, one for each selected attribute, were used to rank the declared preferences of the whole sample (using the standard deviation as an indicator of the preference variability of the whole sample), while the containing the preferences index for each individual was used in the correlation analysis. This analysis was then performed considering the preferences index for each milk attribute, the SD characteristics (gender, age, income, education, and presence of children), and the place of milk purchase (supermarket, convenience stores, discounts, and open market/producer) as variables. It is worth noticing that gender, the presence of children, and all the variables representing the milk purchase place were measured on a dichotomous scale (i.e., Yes/No). PH variables were not mutually exclusive categories and for this reason, participants were able to indicate more than one purchase point. Then, several BW analyses were performed considering the significant variables that emerged from the correlation analysis. Starting from the total sample and the preferences indexes (calculated from each respondent for the single attributes), the ARSs (dependent variable) were compared by clustering the sample in sub-groups using SD characteristics and PH independent variables as discriminating factors. Lastly, we conducted a series of ANOVAs to verify significant differences in SD characteristics (gender, age, education, average annual family income, and milk purchase place) considering each milk attribute as a DV. The between-subject factors were categorized following these parameters: (1) Gender (Man or Woman), (2) Age (a. 18–25, b. 26–35, c. 36–45, d. 46–55, e. > 65), (3) Education (a. primary school, b. lower secondary school, c. upper secondary school, d. master’s degree), (4) Average annual family income (< 25000, 25000–40000, 40000–60000, > 60000), (5) Milk purchase place (a. supermarket, b. convenience store, c. discount, d. open-air market/producer). We conducted a *post hoc* analysis using the Bonferroni correction.

## 3. Results

### 3.1. Sample characteristics

A total of 1,338 participants were recruited but only responses from 1,216 subjects were considered in the analysis as they came from milk purchasers who had completed the questionnaire in its entirety. Of the respondents, 95% consumed milk in addition to buying it. Observing the peaks highlighted in Supplementary Figure 1, the overall sample picture is characterized by the prominence of women, employed, or retired, belonging to a household without dependent children, with a medium-high age (mean = 52 years), a medium-high level of education, and an average annual income.

### 3.2. Cow’s milk preferences

As reported in Table 3, the most important attribute for the choice of cow’s milk is the milk’s origin (ARS = 1.951), followed by the local origin (ARS = 1.500), and the expiry date (ARS = 1.007). On the contrary, the less relevant attributes for milk choice (with a negative AR-S, i.e., the number of times it was chosen as the “worst” exceeded the number of times it was chosen as the “best”) were the packaging material, the organic certification and the fat content (ARS equal to –1.573, –1.374, and –1.178, respectively).

**TABLE 3:** Times selected best, times selected worst, and average raw score (ARS) for single attribute and attributes’ category are reported.

Attributes category	Milk attributes	Times selected best	Times selected worst	ARS	SD	Mean ARS for attribute category
Credence attributes	Organic certification	478.0	1437.0	-1.374	1.995	
	Quality certification	1170.0	475.0	0.925	1.407	0.278
	Local origin	1340.0	564.0	1.500	1.574	
Intrinsic attributes	Fat content (skimmed, semi-skimmed, whole milk)	686.0	1516.0	-1.178	2.422	0.038
	Nutritional value	536.0	994.0	-0.610	1.193	
	Taste	741.0	904.0	-0.265	1.770	
Extrinsic attributes	Packaging material	433.0	1625.0	-1.573	1.724	
	Label information	484.0	1070.0	-0.781	1.238	0.242
	Milk country of origin	1764.0	344.0	1.951	1.913	
	Expiration date	1282.0	476.0	1.007	1.785	
	Price	860.0	785.0	0.158	1.605	
	Brand	1278.0	862.0	0.689	2.463	

### 3.3. Correlations between SDs, PH, and cow’s milk attributes

The correlation analysis showed a different pattern of relationships between the attribution of importance to cow’s milk features, socio-demographic characteristics, and purchasing habits (Table 4). For example, the educational level was positively associated with milk organic certification (*r* = 0.13, *p* < 0.01), taste (*r* = 0.09, *p* < 0.01), and information on the label (*r* = 0.09, *p* < 0.01); while the same SD characteristics were negatively significantly correlated with brand (*r* = 0.10, *p* < 0.01), price (*r* = –0.08, *p* < 0.01), and packaging material (*r* = –0.06, *p* < 0.01). Age was associated positively only with brand (*r* = 0.08, *p* < 0.01) and negatively with organic certification (*r* = –0.09, *p* < 0.01), taste (*r* = –0.07, *p* < 0.01), and information on the label (*r* = –0.09, *p* < 0.01). Among buyers in supermarkets (*n* = 1129), there was a positive correlation with fat content (*r* = 0.11, *p* < 0.01) and a negative correlation with organic certification (*r* = –0.11, *p* < 0.01), quality certification (*r* = –0.07, *p* < 0.01), and information on the label (*r* = –0.08, *p* < 0.01). Among buyers in convenience stores (*n* = 300), there was a positive correlation with quality certification (*r* = 0.12, *p* < 0.01) and information on the label (*r* = 0.08, *p* < 0.01), and a negative correlation with fat content (*r* = –0.14, *p* < 0.01) and nutritional value (*r* = –0.08, *p* < 0.01). Among buyers in discounts (*n* = 59), there was a positive correlation with price (*r* = 0.12, *p* < 0.01). Among buyers in open markets/producers (*n* = 70), there is a positive correlation with local origin (*r* = 0.11, *p* < 0.01) and information on the label (*r* = 0.07, *p* < 0.01) and a negative correlation with brand (*r* = –0.07, *p* < 0.01). In contrast, the presence of children in the family, with a frequency of “yes” equal to 418, did not influence the different preference indexes of the selected attributes.

**TABLE 4:** Correlations (Pearson’s *r*) between preference indices of milk attributes and consumers’ SD characteristics.

	Price	Organic certification	Fat content	Expiration date	Taste	Packaging material	Quality certification	Local origin	National origin	Brand	Information on the label	Nutritional value
Educational level	-0.073*	0.129**			0.091**	-0.061*			-	-0.097**	0.090**	
Average family revenue				-0.071*	-0.064*							
Age		-0.087**			-0.067*					0.077**	-0.081**	
Gender		-0.061*										
Presence of children												
Number of children												
Supermarket		-0.111**	0.109**				-0.074*				-0.094**	
Convenience stores			-0.140**				0.116**				0.079**	-0.071*
Discounts	0.123**											
Open market/producer								0.108**		-0.073*	0.072*	

**p < 0.01; *p < 0.05.

### 3.4. Attribute preference indices in relation to SD characteristics and PH

The one-way ANOVAs, run for detecting differences in purchasers’ preferences related to SD characteristics such as gender, age, education level, and family income, produced the following results.

Considering the individuals’ gender, men (*n* = 387) and women (*n* = 829) showed similar preference indices regarding product attributes. In particular, both considered characteristics such as cow’s milk’s local and national origin as the most important product choice attributes, followed by brand (men with a greater preference) and expiration date (women with a greater preference). In contrast, packaging material and organic certification were the least preferred attributes for both men and women. The only significant differences were the following: fat content [*F*(1, 1214) = 6.06, *p* = 0.014, partial η^2^ = 0.005] and information on the label [*F* (1, 1214) = 3.34, *p* = 0.07, partial η^2^ = 0.003]. Women showed a higher preference for fat attributes, while men showed a higher preference for information labels (Supplementary Figure 2).

Comparing the AR-S clustering of the individuals in age groups, some differences emerged in milk attribute preference indices. The ANOVA showed the following significant differences: price [*F*(5, 1210) = 2.54, *p* = 0.03, η^2^ = 0.010], organic certification [*F*(5, 1210) = 2.48, *p* = 0.03, η^2^ = 0.010], expiry date [*F*(5, 1210) = 3.01, *p* = 0.01, η^2^ = 0.012], national origin [*F*(5, 1210) = 2.29, *p* = 0.04, η^2^ = 0.010], and information on the label [*F*(5, 1210) = 3.50, *p* = 0.00, η^2^ = 0.01] (Supplementary Figure 3). All the considered age groups declared the national origin of the product as the preferred attribute. However, analyzing the results of the B-W clustering, we noticed that purchasers aged 18–25 considered price and brand the less important attribute. Young individuals (aged 18–25, 26–35) considered the taste as an important attribute for milk choice, while the brand was considered positively by subjects aged over 65 and by those in the 36–45 range. Finally, purchasers over 65 and those belonging to the age range 36–45 especially considered the organic certification and the information on the label as not important for milk choice. Concerning the *post hoc* analysis results, we found the following significant differences: price was considered the most positively by people aged 46–55 in comparison with those aged more than 65 years; organic certification was valued differently by people aged 36–45 in comparison with those aged 56–65; expiring date was valued differently by people aged 36–45 in comparison with those aged 56–65; national origin was valued differently by people aged 56–65 in comparison with those aged over 65 and for the latter, this attribute was very important; information on the label was valued differently by people aged 56–65 in comparison with the ones aged over 65 and for the latter, this attribute was not so important (Supplementary Figure 3).

When clustering the purchasers according to education level, the national origin of the product emerged as the most differentiating attribute in the milk decision-making process, followed by the local origin and the expiration date. The less preferred attributes were the organic certification of the product, especially for people who attended primary school, and the packaging material, especially for individuals with master’s degrees. The only significant differences were the following: brand [*F*(3, 1212) = 4.23, *p* = 0.01, η^2^ = 0.010], organic certification [*F*(3, 1212) = 7.51, *p* < 0.001, η^2^ = 0.018], taste [*F*(3, 1212) = 3.62, *p* = 0.01, η^2^ = 0.010], and label [*F*(3, 1212) = 4.48, *p* = 0.04, η^2^ = 0.01]. Participants who attended primary school and lower secondary school considered milk taste as more important in comparison to graduated subjects; the organic certification was considered much less important by individuals with an elementary license than by subjects with high school and university degrees. Information on the label was considered less important by individuals with a primary school education with respect to individuals who graduated and with an upper secondary school certificate. The brand was considered the most positive by people who attended primary school and lower secondary school in comparison with the other categories (Supplementary Figure 4).

As observed for the other clusters created according to socio-demographic characteristics, and also according to the average annual family income, the groups showed a greater preference for the national origin of the product. It is worth noting that 256 participants did not report their income, so the final analysis was run on 960 respondents. Interpreting the results of the B-W approach, we can observe that subjects with a lower income were the most attentive to price and expiry date, while those with the highest income were focused on the national and local origin of the product. The latter also did not consider the fat content of milk as an important factor in their choice (Supplementary Figure 5). The ANOVA showed the following significant differences: organic certification [*F*(3, 956) = 4.51, *p* = 0.00, η^2^ = 0.014], taste [*F*(3, 956) = 3.38, *p* = 0.05, η^2^ = 0.010], brand [*F*(3, 956) = 9.02, *p* < 0.001, η^2^ = 0.03], national origin [*F*(3, 956) = 3.36, *p* = 0.05, η^2^ = 0.010]. Organic certification and taste were considered the most negatively by participants with an average income of 40,000–60,000 compared to the ones earning more than 60,000; while brand and national origin were considered the most positive by the same target in comparison with purchasers earning more than 60.000.

As concerns PH, the one-way ANOVAs performed showed that the choice of the milk purchase place significantly affects the importance given to each selected quality attribute, except in the case of the certification. The significant differences that emerged from the ANOVA are reported in Supplementary Figure 6. Buyers of cow’s milk at discounts were the most price-conscious compared to other subjects (who buy in convenience stores and, above all, in open-air markets/producers) that considered this attribute with a negative preference index. The latter showed a high preference index for the national and local origin of cow’s milk, as well as for the product taste, but not for the producer brand; moreover, they were the only group that showed a positive index for label information (claims) and organic certification. Regular shoppers in supermarkets or convenience stores considered the national and local origin and the expiry date of the product as important attributes. Milk fat content was a non-significant attribute for all groups surveyed (Supplementary Figure 6). From the *post hoc* analysis, we found the following significant results: price was considered positively by purchasers at discount store in comparison to the buyers at open-air market or directly from the producers; taste was evaluated especially positively by the consumers who buy milk directly from the producers in comparison to the buyers at discounts; organic certification was valued differently by people loyal to the milk producers in comparison to the other categories; expiring date was valued with higher importance by purchasers at open-air markets/producers in comparison to the discount and supermarkets buyers; brand was less important for buyers at open-air markets in comparison to discount purchasers; the milk origin, both local and national, were attributes more important for buyers at open-air markets or directly from the producers in comparison to those linked to supermarkets or discount; finally, the label information was evaluated as more important for purchasers at open-air markets in comparison to discount and supermarket purchasers.

## 4. Discussion

This research explored the preferences of cow’s milk purchasers toward different qualities, assessing if and how the individuals’ SD and purchasing habits affect the level of importance given to each attribute describing milk during the decision-making process. In general, our results showed that the perceived quality of a food product depends on the individual with whom it is interfacing. Therefore, purchasers’ perception of milk quality depends not only on their evaluation of the objective characteristics (intrinsic and extrinsic attributes) of the product but also simultaneously on their evaluation of the belief attributes (category with the highest average ARS), as well as their purchasing characteristics and habits. Looking at the ranking of preferences of the entire sample, the perception of quality extends to personal needs, such as the search for a long-lasting product in the larder (attention to the use-by date), and for a product of higher quality (certified), as well as a product from a location close to the place of residence (53–56). In (16), differences were found in the evaluation of the expiry date for the choice of milk; while fresh pasteurized milk consumers pay attention to this attribute for the evaluation of the freshness of the product, UHT milk consumers evaluate the shelf life of the product for the design of a home stock.

Among the SD characteristics, the presence of children, age, income, and education were significantly related to the definition of preferences for some milk attributes (both linked to credence and intrinsic/extrinsic characteristics of the product). It seems that SD variables especially affected the evaluation of intrinsic (for example, taste and fat content) and extrinsic milk attributes while having a lower influence on the evaluation of credence attributes, partially confirming the first hypothesis (H1). In fact, only income and the point of milk purchase determined significant differences in local production, certification, and organic milk preferences. However, this latter finding allows us to confirm the second research hypothesis (H2: PH affects the decision of cow’s milk purchase).

The results suggest that socio-demographic variables have relatively weak explanatory power relative to the attitudes of individuals toward credence attributes, in accordance with the existing literature (57, 58). In fact, as suggested by some studies (59, 60), the socio-psychological (e.g., values, norms, and beliefs) and behavioral variables are the most important individual characteristics in explaining purchase intention for credence attributes. At the same time, however, different studies showed that women are more sensitive toward ethical and environmental concerns linked to animal production (61, 62). In contrast, in this study, gender did not seem to determine differences in the milk attributes evaluation.

In parallel, different consumption profiles were defined including the place of purchase as a discriminating factor. For example, the greater importance of local production and product taste for purchasers who choose milk at farmers’ markets or directly from producers highlighted the positive correlation between the local product and product typicality and quality, as well as the recognition of the value of the short supply chain (26, 63, 64) (H2 is acceptable). The spread in recent years of new forms of organizing food distribution, such as solidarity purchasing groups, direct sales, and farmers’ markets, could be linked to the increasingly important role played by belief attributes in shaping purchaser preferences toward certain products, such as fruit and vegetables, but also products of animal origin, such as milk and dairy products. Indeed, the increasing popularity of these distribution channels allows consumers to support local agriculture and, at the same time, to purchase higher quality foods to which they attribute higher levels of safety (26, 54, 55). However, while local production is highly promoted at the point of sale of large retail chains, the consumer seems to perceive this characteristic only in the case of purchasing from the producer, showing clear profiling by consumers.

At the same time, individuals that purchase milk at a supermarket considered milk price and brand as the most important attributes in their decision-making process, basing their choice on product convenience and the habit and tradition (previous knowledge) of choosing a specific brand. The difference in preferences toward individual attributes of choice by comparing shopping locations precludes the importance of planning assortment choices and communication campaigns in different shop formats (39). It could therefore be important to properly communicate the value (e.g., local branding) and the higher quality of the products obtained from these local systems as differentiation tools in the market to increase transparency and create greater consumer awareness. Consumer awareness might be increased using product label information. This information can influence consumers’ preferences, behaviors, and willingness to pay, especially in the case of certifications (65–68).

Product purchase place has an important role and influences the interpretation of label information. In fact, except for the consumer that buys milk at the open-air market, the label was always an unimportant attribute for milk choice. Even if the label is very important, by becoming a tool that can affect the purchaser’s food choices (69), it continues to be an inefficient tool for conveying information to the consumer. Probably, in the case of milk, the product information that is important for purchasers is not the intrinsic information known for a product such as milk, but rather those aspects that are useful for product differentiation, such as the production process, the added value linked to nutritional aspects [contribution in mountain milk (CLA)] (15), and the environmental and social sustainability of the supply chain (70), communicated using the brand and corporate values (66, 71).

## 5. Conclusion

This work allowed us to define differences in purchasers’ preferences toward a set of heterogeneous attributes of cow’s milk by comparing different profiles of individuals defined by their socio-demographic characteristics and purchasing habits. The results revealed different milk choice orientations defined mainly by the choice of sales channels and certain SD characteristics such as income and age. The differentiated evaluation of product descriptors is necessary to optimize the valorization and recognition of the product in the market. Interestingly, the milk belief variables, which are among the most important for milk choice, are little influenced by socio-demographic characteristics but vary significantly according to purchasing locations. Therefore, research hypothesis H1 is only partially fulfilled, precluding future developments of this research that will explore how behavioral characteristics, norms, and beliefs of individuals affect consumers’ stated preferences. In contrast, hypothesis H2 is confirmed.

This paper contributes to the literature with concrete implications for production, marketing, and target-driven milk value decisions. Moreover, given that the attributes also included nutritional characteristics of the product, such as fat content, this work makes it possible to identify consumption profiles of those whose choices are dictated by nutritional parameters (also akin to supporting healthy eating styles, which are becoming increasingly popular among the modern consumer), favoring strategies to improve both the product itself and its communication. Given the research was limited to two regions in Northwest Italy, we will have to investigate preferences more broadly in the future by considering other geographical areas and extending the analysis of variables (both behavioral and related to individual lifestyle) describing individuals. These findings emphasize the need for comprehensive research on the definition of buyer preferences that will enable the design of products tailored to consumer needs and the effective communication of attributes that are the most influential on choice according to different consumer targets and needs.

## Data availability statement

The raw data supporting the conclusions of this article will be made available by the authors, without undue reservation.

## Ethics statement

The studies involving human participants were reviewed and approved by University Bioethics Committee of the University of Turin. The patients/participants provided their written informed consent to participate in this study.

## Author contributions

VM and OM: conceptualization, methodology, and formal analysis. VM and SB: data curation. VM, GM, OM, and FF: writing—original draft preparation. VM, SM, SB, DB, AS, and FF: writing—review and editing. SM, SB, DB, OM, and FF: visualization. VM, SM, and DB: supervision. All authors read and agreed to the published version of the manuscript.

## Footnotes

1 https://www.unito.it/ricerca/strutture-e-organi-la-ricerca/comitato-di-bioetica-dellateneo/, accessed on 1 September 2022.

2
http://www.sawtoothsoftware.com/
