# Supplementary material for: The role of socio-demographic variables and buying habits in determining milk purchasers’ preferences and choices
Source: Front Nutr. 2023 Feb 8;10:1072208. doi: 10.3389/fnut.2023.1072208 (PMC9944046; doi:10.3389/fnut.2023.1072208)
Supplement: Supplementary file 1 [file Data_Sheet_1.PDF]

Figures

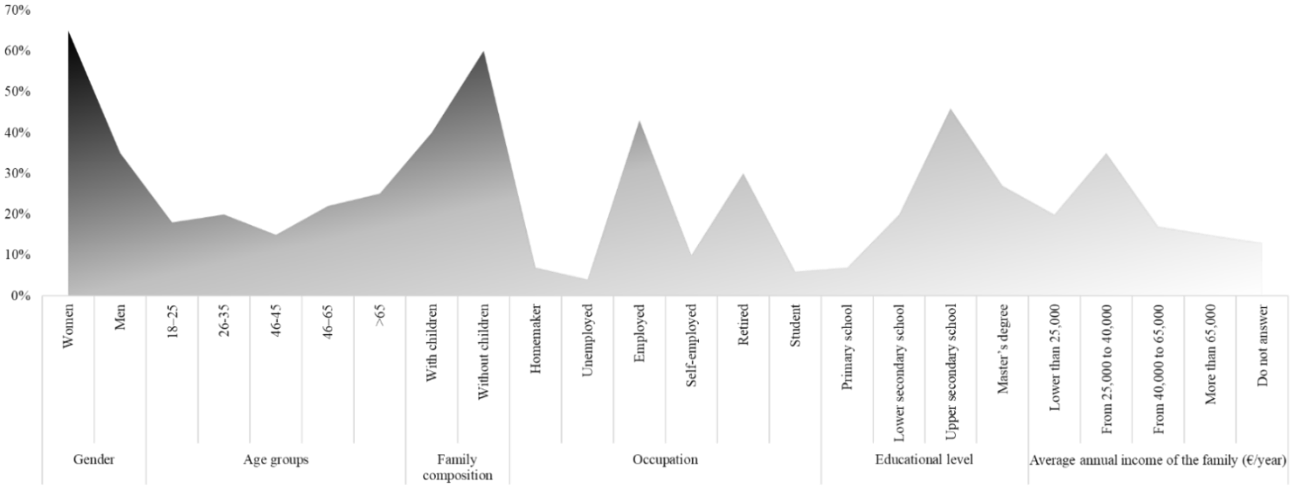

Figure 1 Socio-demographic profile of the sample.

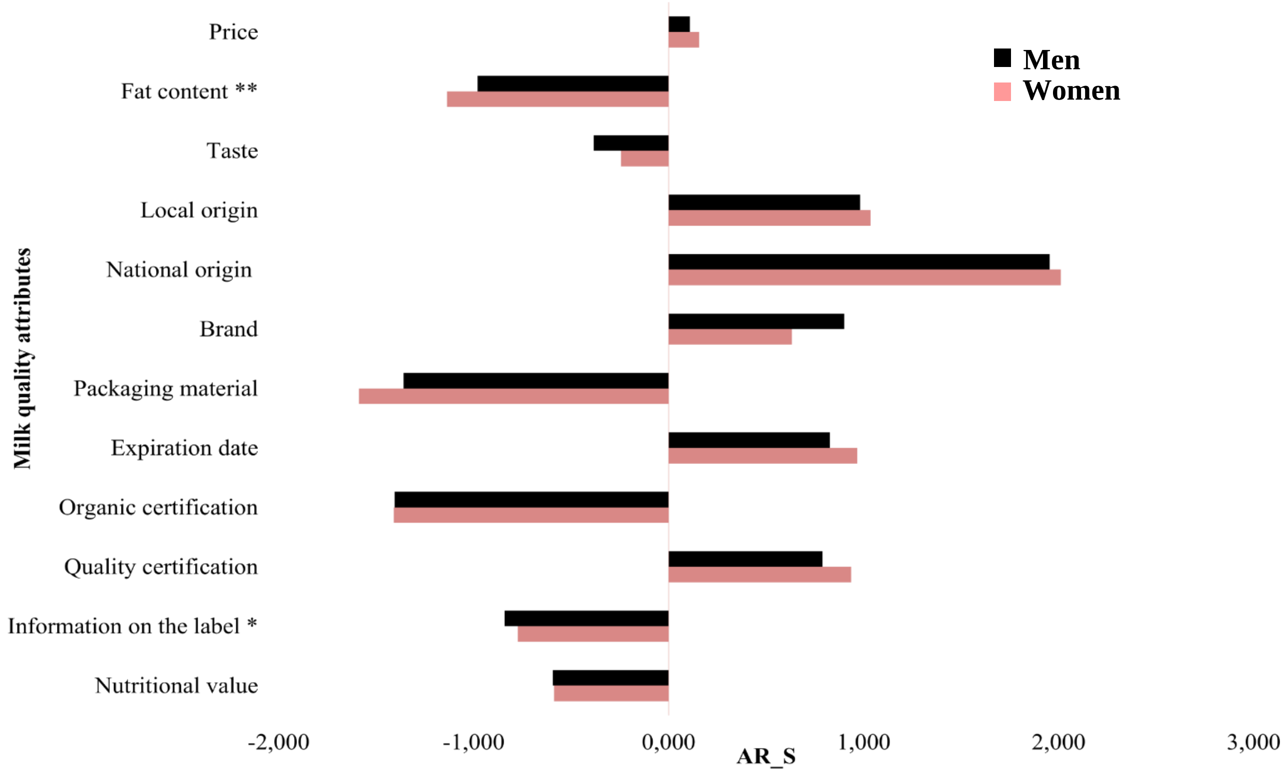

Figure 2 AR-S calculated for each cow milk attributes considering the consumers' gender. **Note:** ** *p*<.01; * *p*<.05

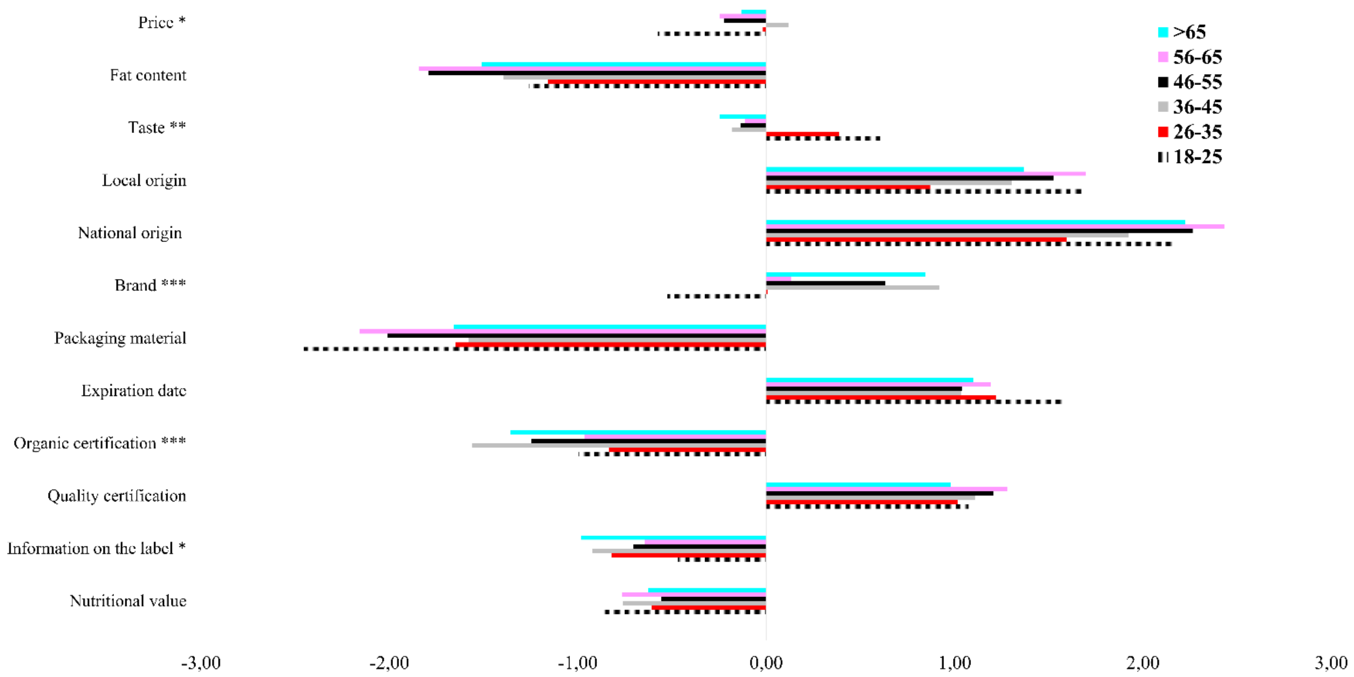

Figure 3 AR-S calculated for each cow milk attributes considering the consumers' age. **Note:** ** *p*<.01; * *p*<.05

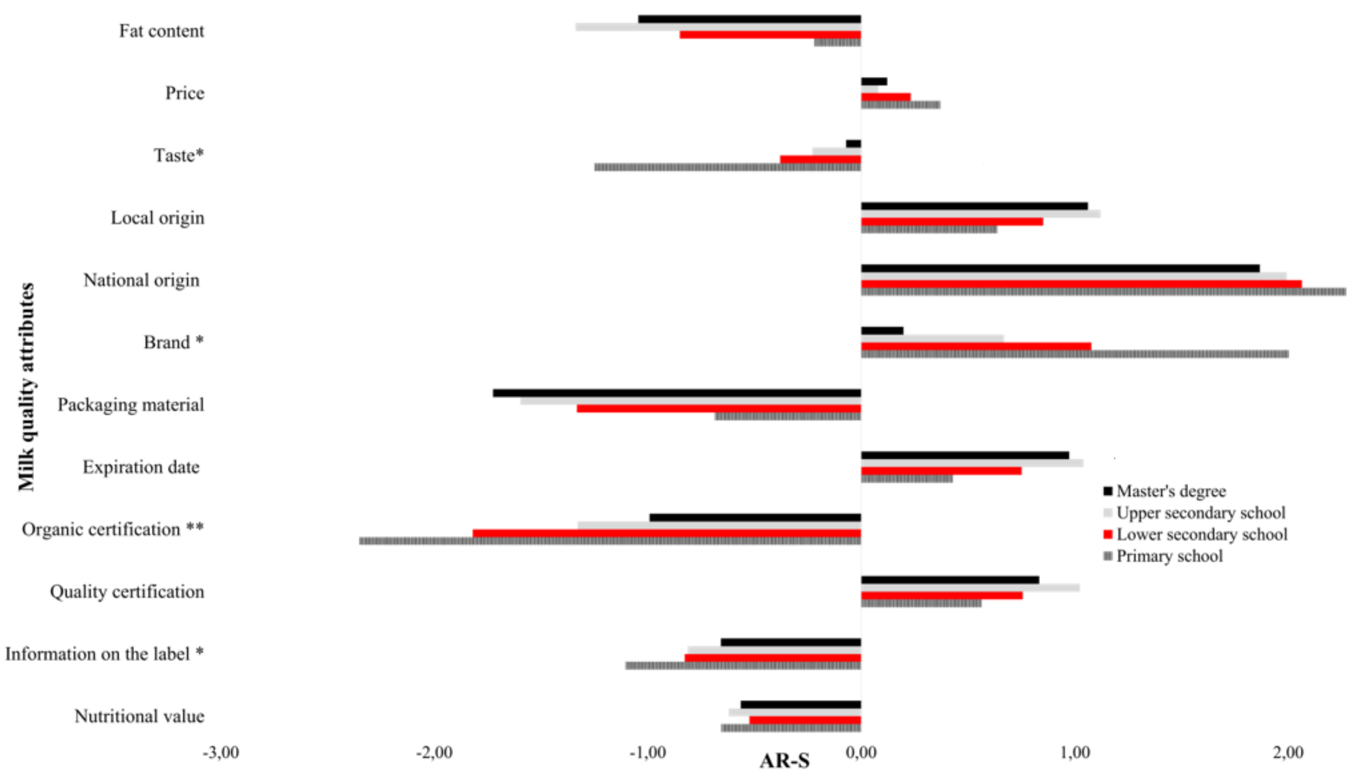

Figure 4 AR-S calculated for each cow milk attributes considering the consumers' educational level.

Note: ** *p*<.01; * *p*<.05

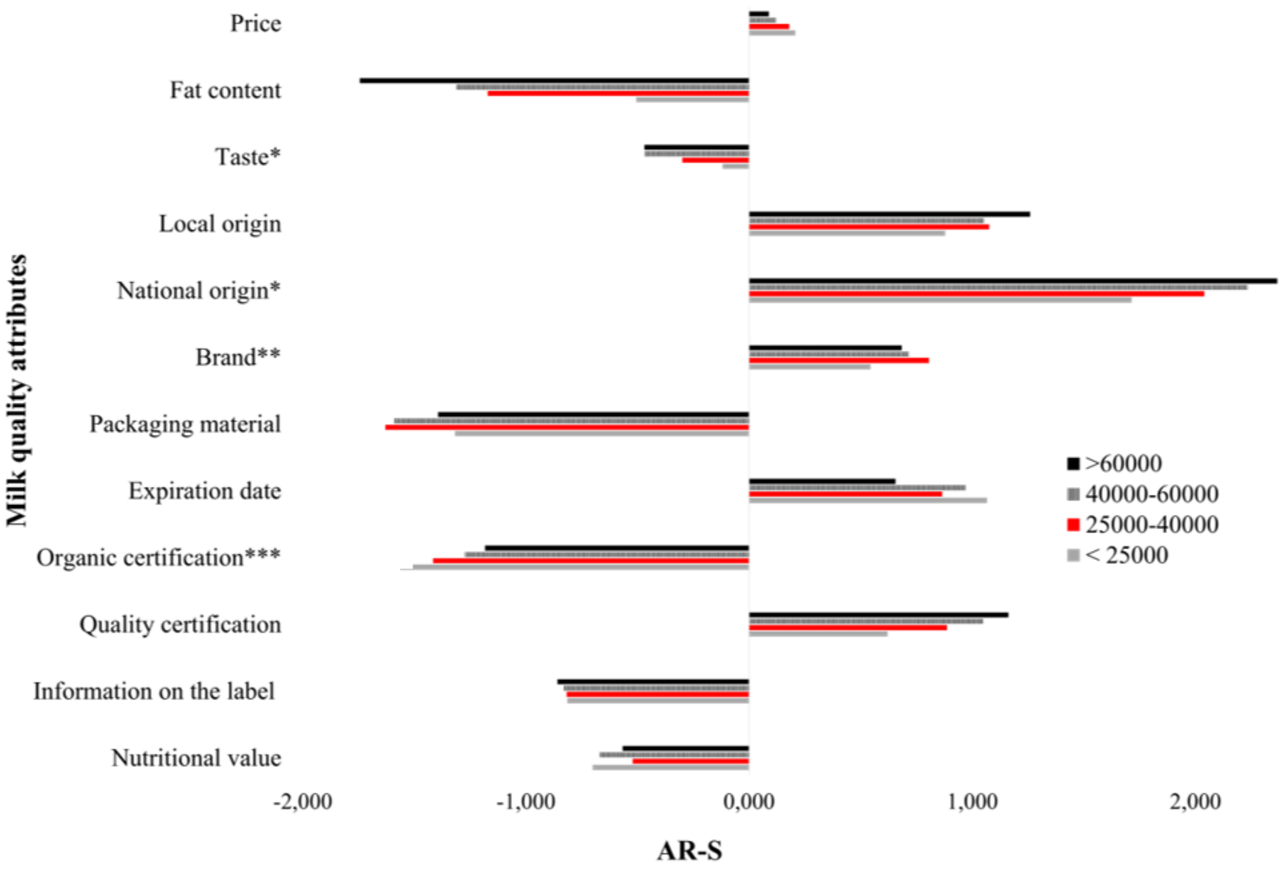

Figure 5 AR-S calculated for each cow milk attributes considering the consumers' average annual income. **Note:** ** *p*<.01; * *p*<.05

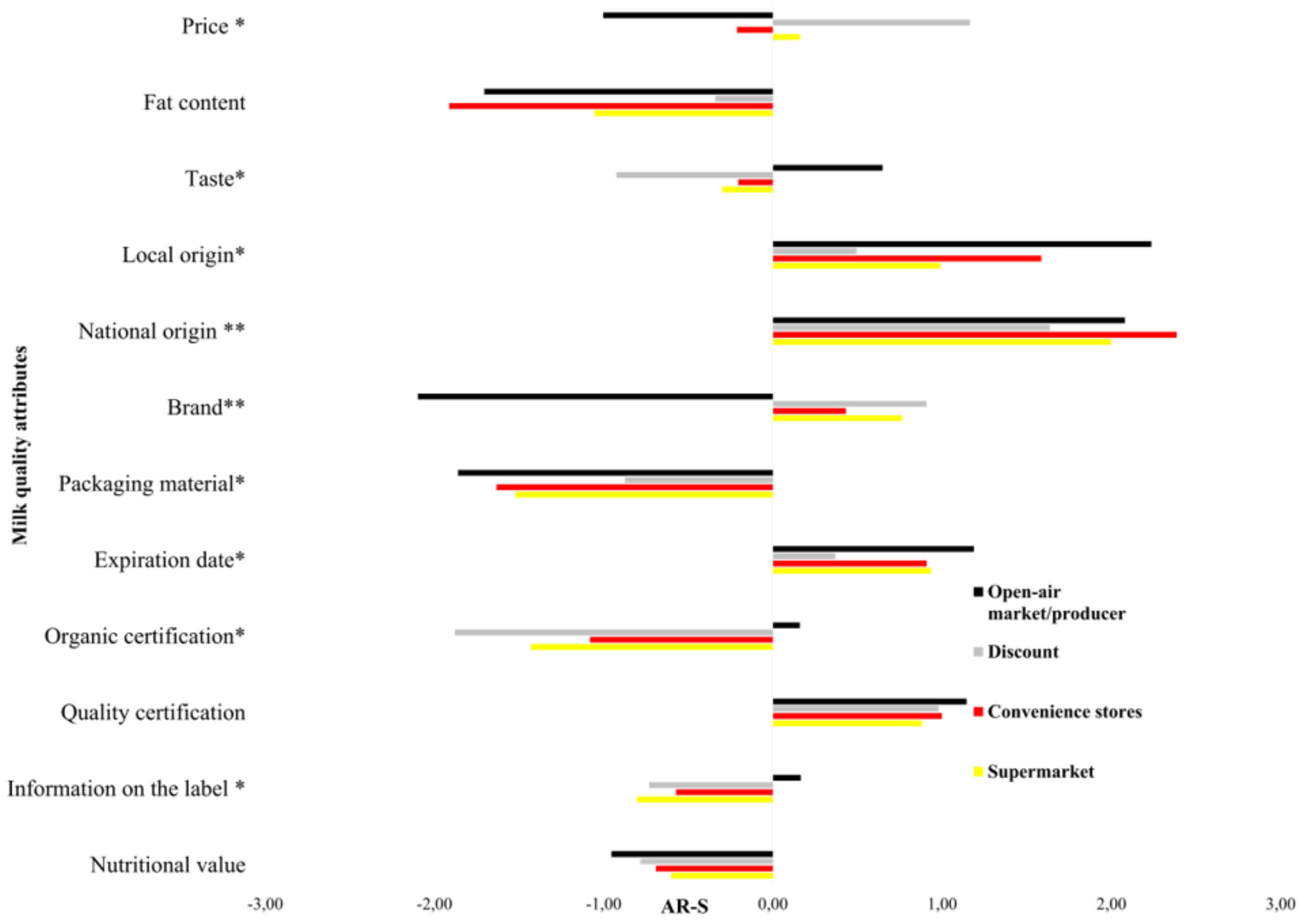

Figure 6 Preference indices (ARSs) of cow milk attributes analyzed considering the consumers' purchasing habits (points of milk purchase). **Note:** ** p<.01; * p<.05
