# Supplementary material for: The role of socio-demographic variables and buying habits in determining milk purchasers’ preferences and choices
Source: Front Nutr. 2023 Feb 8;10:1072208. doi: 10.3389/fnut.2023.1072208 (PMC9944046; doi:10.3389/fnut.2023.1072208)
Supplement: Supplementary file 2 [file Data_Sheet_2.PDF]

Supplementary materials

Example of a questionnaire version employed in the data collection (translated).

SOCIO-DEMOGRAPHIC CHARACTERISTICS

- Age >=18
  - Yes
  - No
- Do you buy milk for yourself or are you the milk buyer in your family?
  - Yes
  - No

Eligibility criteria

1. Please, specify your age:
   - 18-25
   - 26-35
   - 36-45
   - 46-55
   - 65

2. Gender
   - Men
   - Woman
   - I prefer not to answer

3. How many people are in your family _______
4. Occupation:
   - student
   - employed
   - self-employed
   - retired
   - looking for work
   - housewife/homemaker
   - other:______________________________
5. Educational level
   - Primary school
   - Lower secondary school
   - Upper secondary school
   - Master's degree
6. What is the average annual income of your household?
   - lower than €25,000
   - between €25,000 and €40,000
   - between €40,000 and €60,000
   - greater than €60,000
7. Do you consume milk?

- o yes
- o no

COW'S MILK PURCHASING HABITS

8. What type of cow's milk do you buy?
    - o Fresh pasteurized
    - o UHT (long shelf life)
    - o None
9. Usually, where do you buy cow milk: Milk purchase place (a. supermarket, b. convenience store, c. discount, d. open-air market/producer).
    - o supermarkets/hypermarkets
    - o convenience stores
    - o open-air markets/producer
    - o discount

BEST-WORST SCALING QUESTIONNAIRE: CONSUMER PREFERENCE SURVEY

Try to remember the last time you purchased cow's milk.
Indicate the most important (BEST) (only one choice) and the least important (WORST) (only one choice) attributes during the milk choice:

MOST IMPORTANT (one answer)	CHARACTERISTIC	LEAST IMPORTANT (one answer)
o	Price	o
o	Organic certification	o
o	Fat content (skim, partially skimmed, and whole)	o
o	Expiry date	o

Indicate the most important (BEST) (only one choice) and the least important (WORST) (only one choice) attributes during the milk choice:

MOST IMPORTANT (one answer)	CHARACTERISTIC	LEAST IMPORTANT (one answer)
o	Taste	o
o	Package type (plastic jug, cardboard carton, glass)	o
o	High-quality certification	o
o	Origin indication (national/abroad)	o

Indicate the most important (BEST) (only one choice) and the least important (WORST) (only one choice) attributes during the milk choice:

MOST IMPORTANT (one answer)	CHARACTERISTIC	LEAST IMPORTANT (one answer)
o	Locally farmed	o
o	Brand	o
o	Label claims (visual and verbal)	o
o	Nutritional value	o

Indicate the most important (BEST) (only one choice) and the least important (WORST) (only one choice) attributes during the milk choice:

MOST IMPORTANT (one answer)	CHARACTERISTIC	LEAST IMPORTANT (one answer)
o	Package type (plastic jug, cardboard carton, glass)	o
o	Price	o
o	Nutritional value	o
o	Organic certification	o

Indicate the most important (BEST) (only one choice) and the least important (WORST) (only one choice) attributes during the milk choice:

MOST IMPORTANT (one answer)	CHARACTERISTIC	LEAST IMPORTANT (one answer)
○	Brand	○
○	Taste	○
○	Price	○
○	Locally farmed	○

Indicate the most important (BEST) (only one choice) and the least important (WORST) (only one choice) attributes during the milk choice:

MOST IMPORTANT (one answer)	CHARACTERISTIC	LEAST IMPORTANT (one answer)
○	Expiry date	○
○	Label claims (visual and verbal)	○
○	Brand	○
○	Package type (plastic jug, cardboard carton, glass)	○

Indicate the most important (BEST) (only one choice) and the least important (WORST) (only one choice) attributes during the milk choice:

MOST IMPORTANT (one answer)	CHARACTERISTIC	LEAST IMPORTANT (one answer)
○	Expiry date	○
○	Label claims (visual and verbal)	○
○	Fat content (skim, partially skimmed, and whole)	○
○	Taste	○

Indicate the most important (BEST) (only one choice) and the least important (WORST) (only one choice) attributes during the milk choice:

MOST IMPORTANT (one answer)	CHARACTERISTIC	LEAST IMPORTANT (one answer)
○	High-quality certification	○
○	Fat content (skim, partially skimmed, and whole)	○
○	Origin indication (national/abroad)	○
○	Locally farmed	○

Indicate the most important (BEST) (only one choice) and the least important (WORST) (only one choice) attributes during the milk choice:

MOST IMPORTANT (one answer)	CHARACTERISTIC	LEAST IMPORTANT (one answer)
○	Nutritional value	○
○	Origin indication (national/abroad)	○
○	Organic certification	○
○	High-quality certification	○

The questionnaire has ended!

Thank you for taking a few minutes to do the research

Best regards!
